# Immunohistochemical Evaluation of S100, Alpha-Smooth Muscle Actin, Podoplanin, Matrix Metalloproteinase 13, and Human Epidermal Growth Factor Receptor 2neu Markers in Basal Cell Carcinoma Variants

**DOI:** 10.7759/cureus.31221

**Published:** 2022-11-07

**Authors:** Shatha A Jabour, Ban F Al-Drobie, Bashar H Abdullah, Ameer D Hameedi

**Affiliations:** 1 Oral and Maxillofacial Pathology, College of Dentistry, University of Baghdad, Baghdad, IRQ; 2 Pathology, College of Medicine, University of Baghdad, Baghdad, IRQ

**Keywords:** head and neck, immunohistochemistry, her2neu, mmp-13, podoplanin, α-sma, s100, basal cell carcinoma variants

## Abstract

Background

Variants of basal cell carcinoma (BCC) appear to behave biologically differently. Several histological patterns impact the concept of low-risk (indolent) and high-risk (aggressive) types in the head and neck. This study aims to assess the biological behavior of BCC variants by immunohistochemical expression of S100, alpha-smooth muscle actin (α-SMA), podoplanin, matrix metalloproteinase 13 (MMP-13), and human epidermal growth factor receptor 2 (HER2)neu biomarkers.

Methodology

A total of 65 paraffin-embedded tissue blocks of BCC of the head and neck were retrieved from the collections of the Histopathology Department of the Medical City Teaching Complex and the Ghazi Al-Harerri Hospital at the University of Baghdad’s College of Dentistry, spanning the years 2015 through 2021. S100, α-SMA, podoplanin, MMP-13, and HER2neu biomarkers were used to perform immunohistochemical analysis (Abcam).

Results

This study noticed different expressions of S100, α-SMA, podoplanin, MMP-13, and HER2neu between different variants. There was no immunohistochemical expression in perineural invasion with all cases of BCC variants. The highest expression was seen in HER2neu, MMP-13, and α-SMA with aggressive histological patterns. There was no podoplanin lymphatic vessel density immunoexpressing in all variants, while tumoral podoplanin showed a significant difference in all variants. HER2neu was correlated with all other biomarkers.

Conclusions

HER2neu, MMP-13, and α-SMA biomarkers can be used as diagnostic markers to predict the aggressive biological behavior of BCC tumors.

## Introduction

The malignant skin tumor known as basal cell carcinoma (BCC) is composed of clusters of basal cells. It has various clinical presentations based on the presence of different morphological features that, to some extent, coincide with the different histological types. Variants of BCC appear to exhibit distinct biological behavior [1,2]. It is uncertain if these tumors represent distinct developmental lines or are part of a continuous spectrum of carcinogenesis that ranges from indolent to aggressive forms due to their diverse biological properties.

Numerous studies have lately been conducted to identify new prognostic markers of BCC that play a crucial role in malignant cell transformation processes. Identifying these biomarkers is critical for predicting how cancer will behave in the future [3-5].

Although BCC seldom metastasizes, it often demonstrates localized aggressiveness and has a high risk of local recurrence: 12% on average and as high as 65% in aggressive histologic variants. Clinically meaningful aggression indicators are variable [6,7].

Using basement membrane (BM) molecules, it has been demonstrated that BCC and its stromal component are intimately related and that the interaction between the tumor mass and its stroma is crucial to disease pathogenesis. With the transition from indolently to aggressively growing BCC, loss of BM material occurs around specific tumor cell nests [8].

Further research on BCC has shown that the tumor microenvironment (TME) appears to be significantly influenced by cancer-associated fibroblasts (CAF) displaying various cellular phenotypes. In contrast to nodular BCC, aggressive subtypes of BCC called micronodular and morpheaform have been shown to generate stromal cells with an actin-rich phenotype [9,10].

Podoplanin affects tumor invasion, progression, and lymphatic vessel development. Some cutaneous neoplasms, sebaceous germinative cells, and hair follicle basal layers respond to it [11,12].

Podoplanin expression is linked to cell migration, epidermal to mesenchymal transition (EMT), and a distinct tumor cell invasion mechanism without EMT. Podoplanin is involved in tumor cell actin reorganization and may increase invasion by enhancing cell motility [13,14].

The S100 protein was the first melanocytic-associated marker with the best sensitivity and practical value in melanoma diagnosis and sentinel lymph node (SLN) evaluation. Even though S100 proteins may play important roles in many cancers, it remains unclear if members of the S100 family are expressed in epidermal tumors and linked to carcinogenesis [15,16].

Matrix metalloproteinases (MMPs) are proteolytic enzymes that break down the extracellular matrix. Normal, unbroken skin lacks MMP-13, or collagenase-3, which cleaves collagen, gelatin, and fibronectin. Malignant squamous cell carcinomas and BCCs express MMP-13 [17,18].

Numerous tissues express human epidermal growth factor receptor 2 (HER2), and these tissues primarily function to promote excessive/uncontrolled cell proliferation and cancer. Evidence suggests that epidermal growth factor receptors (EGFRs) cooperate with other processes to promote BCC progression and aggressiveness [19,20].

The purpose of this research is to evaluate the biological behavior and histological subtypes of head and neck BCC by analyzing the expression of biomarkers such as alpha-smooth muscle actin (α-SMA), podoplanin, S100, MMP-13, and HER2neu using immunohistochemistry.

## Materials and methods

Between 2015 and 2021, the Oral and Maxillofacial Pathology Department at the College of Dentistry, University of Baghdad, Ghazi Al-Harerri Hospital, Medical City, and the Histopathology Department, Medical City Teaching Complex retrieved 65 surgically removed, paraffin-embedded tissue blocks of BCC of the head and neck. Patient reports and tumor histology data were obtained from laboratory results. Two different pathologists independently evaluated the histopathology of the tumor. For each case, 4 μm of a selected block was used for immunohistochemistry.

The datasheet for Abcam’s immunohistochemical (IHC) detection kit showed that the samples were deparaffinized in xylene, dried in ethanol, and pretreated with a heat-mediated, antigen-retrieving solution (Micro-polymer ab 23649, USA; Rabbit-specific horseradish peroxidase (HRP)/diaminobenzidine (DAB) detection IHC detection kit). Table 1 lists the antibodies utilized in this investigation along with some of their key characteristics.

**Table 1:** The characteristics of antibodies in the IHC procedure. α-SMA: alpha-smooth muscle actin; MMP-13: matrix metalloproteinase 13; HER2: human epidermal growth factor receptor 2; IHC: immunohistochemical

Antibody	Code/Clonality/Host	Dilution	Buffer	Positive control
S100	Ab136629/monoclonal/rabbit	1/100	Citrate pH: 7.4	Human neurofibroma
α-SMA	Ab265588/monoclonal/rabbit	1/100	Citrate pH: 7.4	Human colon carcinoma
Podoplanin	Ab236529/monoclonal/rabbit	1/4,000	Citrate pH: 7.4	Normal human colon
MMP-13	Ab219620/monoclonal/rabbit	1/350	Citrate pH: 7.4	Human breast carcinoma
Erb2/HER2	Ab16662/monoclonal/rabbit	1/200	Citrate pH: 7.4	Human breast carcinoma

Primary antibodies were placed at room temperature for one hour and rinsed with phosphate-buffered saline (PBS). Subsequently, two to three drops of goat anti-rabbit HRP conjugate were applied to the sections and incubated for 30 minutes at 37°C within the humid chamber. In the last rinse of PBS, the chromogen and DAB substrate were prepared according to the manufacturer’s datasheet by adding one drop of chromogen to 50 drops of DAB substrate.

Hematoxylin counterstain was done by rinsing the slides for three minutes according to the patency of the used hematoxylin, followed by dehydration using ethanol for each slide and, finally, using xylene one to two drops of DPX mounting medium applied to the xylene wet sections and covered with coverslips gently to remove excess air bubbles and then left to dry. Negative tissue controls were used by adding PBS to each slide of any section.

Statistical analysis

Using SPSS version 25 (IBM Corp., Armonk, NY, USA), a chi-square test was done to examine clinical factors. The independent t-test and two-tailed analysis of variance (ANOVA) were used to examine continuous variables. The Pearson correlation test (r) was performed to analyze the relationship between continuous variables. P-values of 0.05 were considered significant.

## Results

Immunohistochemistry of S100

**Figure 1:**
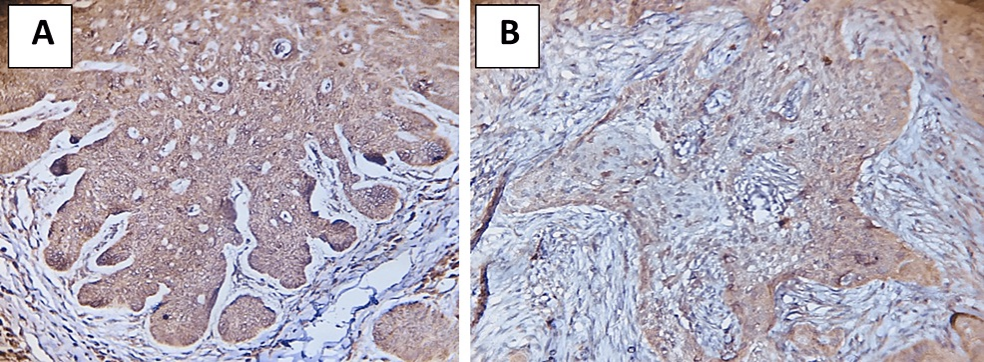
Immunohistochemical evaluation of S100 (A) tumoral cells adenoid (×20) and (B) tumoral cells metatypical (×20).

The tumoral S100 immunoexpression by histopathologic variants was considerably significant (p = 0.037). The mean ± standard deviation (3.0 ± 0) was higher in adenoid and metatypical variants than in the other BCC variants (Table 2, Figures 1A, 1B). Regarding the S100 perineural invasion estimate, all BCC variants lacked IHC assessment.

**Table 2:** Mean of S100 expression in histological subtypes of basal cell carcinoma. P-values are significant at ≥0.05.

Histological type	S100 tumoral cell (T) (mean ± SD)	P-value
Nodular	2.60 ± 0.59	0.037
Micronodular	2.60 ± 0.89	
Adenoid	3.0 ± 0	
Infiltrative	2.80 ± 0.60	
Infundibulocystic	2.80 ± 0.44	
Metatypical	3.0 ± 0	

Expression of alpha-smooth muscle actin by immunohistochemistry

**Figure 2:**
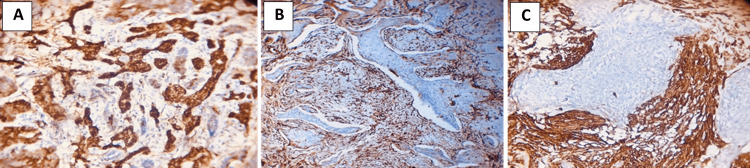
Immunohistochemical evaluation of α-SMA: (A) tumoral cells infiltrative (×20), (B) stromal cells infiltrative (×10), and (C) stromal cells metatypical (×20). α-SMA: alpha-smooth muscle actin

The tumoral immunoreactivity of α-SMA was significantly expressed (p = 0.047) in all BCC variants, and patients with infiltrative BCC had a higher mean (2.57 ± 0.35) compared to other BCC variants. Regarding stromal α-SMA expression, there was a statistically significant difference across BCC subtypes (p = 0.001). The mean was higher in metatypical (3.0 ± 0) and infiltrative (2.71 ± 0.48) than in other BCC variants (Table 3, Figure 2A-2C).

**Table 3:** Mean of α-SMA expression in histological subtypes of basal cell carcinoma. α-SMA: alpha-smooth muscle actin

Histological type	α-SMA tumoral cell (T) (mean ± SD)	α-SMA stromal cells (S) (mean ± SD)	P-value
Nodular	1.70 ± 0.73	2.40 ± 0.75	T = 0.047 S = 0.001
Micronodular	1.60 ± 0.89	1.60 ± 0.54	
Adenoid	2.16 ± 0.83	2.50 ± 0.67	
Infiltrative	2.57 ± 0.53	2.71 ± 0.48	
Infundibulocystic	2.20 ± 1.09	2.60 ± 0.54	
Metatypical	2.31 ± 0.60	3.0 ± 0	

Immunohistochemical expression of podoplanin

**Figure 3:**
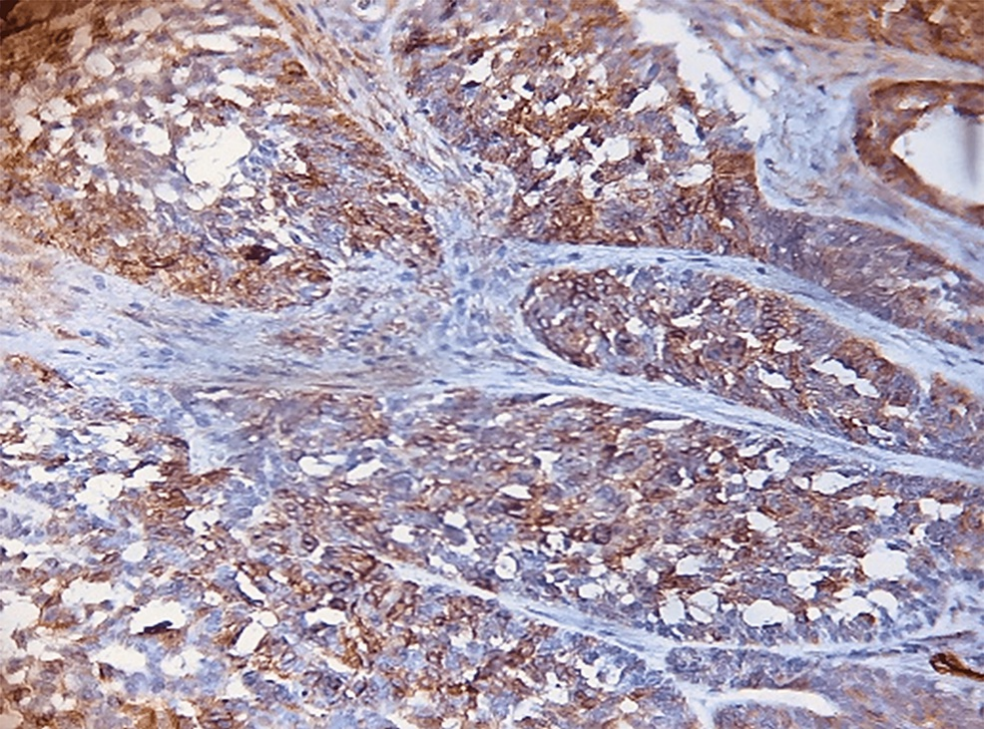
Immunohistochemical evaluation of podoplanin, adenoid tumoral cell (×20).

Regarding tumoral cell podoplanin immunoexpression, BCC variants exhibited a significant difference (p = 0.029). The mean (3.50 ± 3.11) was higher in the adenoid type than in other BCC variants. Regarding podoplanin lymphatic vessel density (LVD), no significant difference was found in all BCC variants (Table 4, Figure 3).

**Table 4:** Mean of podoplanin expression in histological subtypes of basal cell carcinoma. LVD: lymphatic vessel density

Histological type	Podoplanin tumoral cell (T) (mean ± SD)	Podoplanin LVD (mean ± SD)	P-value
Nodular	1.45 ± 0.94	16.90 ± 5.31	T = 0.029 LVD = 0.065
Micronodular	1.20 ± 0.44	18.61 ± 4.71	
Adenoid	3.50 ± 3.11	17.41 ± 4.04	
Infiltrative	1.42 ± 0.53	17.26 ± 6.77	
Infundibulocystic	1.60 ± 0.54	9.99 ± 5.99	
Metatypical	2.81 ± 2.37	20.60 ± 8.85	

Immunohistochemical expression of matrix metalloproteinase 13

**Figure 4:**
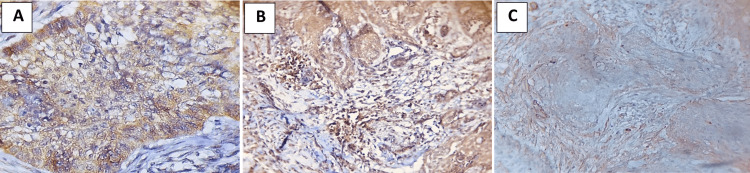
Immunohistochemical evaluation of MMP-13: (A) tumoral cells metatypical (×40), (B) tumoral and stromal cells metatypical (×20), and (C) stromal cells infiltrative (×20). MMP-13: matrix metalloproteinase 13

Regarding tumoral MMP-13, a significant difference was (p = 0.049) noted in BCC variants. The mean was (2.25 ± 0.68) for the metatypical BCC type. Regarding stromal MMP-13 expression, there was a significant difference (p = 0.001) between all BCC variants. The mean was higher for infiltrative (3.00 ± 0) and metatypical BCC (2.18 ± 0.65) variants compared to other variants (Table 5, Figure 4A-4C).

**Table 5:** Mean of MMP-13 expression in histological subtypes of basal cell carcinoma. MMP-13: matrix metalloproteinase 13

Histological type	MMP-13 tumoral cell (T) (mean ± SD)	MMP-13 stromal cells (S) (mean ± SD)	P-value
Nodular	1.40 ± 0.75	1.35 ± 0.48	T = 0.049 S = 0.001
Micronodular	1.80 ± 0.83	1.80 ± 0.44	
Adenoid	1.66 ± 0.88	1.41 ± 0.51	
Infiltrative	1.42 ± 0.78	3.0 ± 0	
Infundibulocystic	1.80 ± 0.83	1.40 ± 0.54	
Metatypical	2.25 ± 0.68	2.18 ± 0.65	

Immunohistochemical expression of human epidermal growth factor receptor 2neu

**Figure 5:**
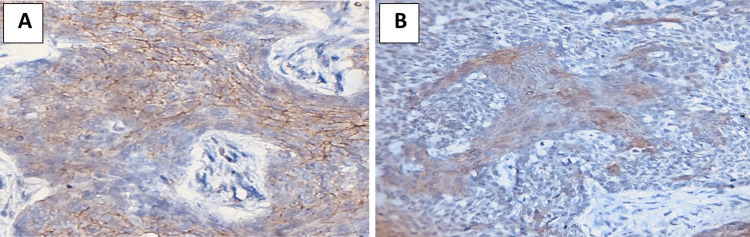
Immunohistochemical evaluation of HER2: (A) infiltrative (×40) and (B) HER2 metatypical (×20). HER2: human epidermal growth factor receptor 2

In histopathologic variants of BCC, HER2neu immunoexpression was very significant (p = 0.001). Metatypical (6.62 ± 2.18) and infiltrative (5.71 ± 3.45) variants showed a higher mean than other BCC variants (Table 6, Figures 5A, 5B).

**Table 6:** Mean of HER2neu expression in histological subtypes of basal cell carcinoma. HER2: human epidermal growth factor receptor 2

Histological type	HER2neu tumoral cell (T) (mean ± SD)	P-value
Nodular	2.05 ± 1.87	0.001
Micronodular	2.20 ± 1.64	
Adenoid	2.50 ± 1.88	
Infiltrative	5.71 ± 3.45	
Infundibulocystic	2.80 ± 1.92	
Metatypical	6.62 ± 2.18	

Correlation between immunohistochemical markers

Significant differences were noted for each of the tumoral S100, tumoral α-SMA, stromal α-SMA, and podoplanin LVD with tumoral HER2neu. Tumoral podoplanin was significantly correlated with tumoral MMP-13 and tumoral HER2neu. Tumoral MMP-13 was significantly correlated with stromal MMP-13 and tumoral HER2neu. There was a significant correlation between stromal MMP-13 and HER2neu. Tumoral HER2neu had a strong correlation with itself (Table 7).

**Table 7:** Correlation between Immunohistochemical markers. α-SMA: alpha-smooth muscle actin; MMP-13: matrix metalloproteinase 13; HER2: human epidermal growth factor receptor 2; LVD: lymphatic vessel density

Markers		Tumoral α-SMA	Stromal α-SMA	Podoplanin (LVD)	Tumoral podoplanin	Tumoral MMP13	Stromal MMP13	Tumoral HER2neu
Tumoral S100	P-value	0.20	0.97	0.85	0.10	0.18	0.54	0.03
Tumoral α-SMA	P-value	__	0.44	0.98	0.64	0.07	0.09	0.01
Stromal α-SMA	P-value	__	__	0.34	0.35	0.82	0.08	0.03
Podoplanin (LVD)	P-value	__	__	__	0.58	0.35	0.38	0.02
Tumoral Podoplanin	P-value	__	__	__	__	0.01	0.91	0.03
Tumoral MMP13	P-value	__	__	__	__	__	0.03	0.04
Stromal MMP13	P-value	__	__	__	__	__	__	0.01
Tumoral HER2neu	P-value	__	__	__	__	__	__	0.01

## Discussion

The BCC categorization is complex and represents a heterogeneous collection of tumors with varying clinical and morphological presentations. This study showed no immunohistochemical perineural invasion, and this finding is explained by one of the major features of BCC, that is, it very rarely metastasizes. This is associated with both increased morbidity and higher recurrence rates [21,22].

This study scored S100 according to the previous study by Zhu et al. [16] and found significant differences between BCC subtypes regardless of low or high-risk histological features, which is corroborated by prior research showing that the S100 protein has a wide variety of intracellular and extracellular functions in vivo [23,24].

IHC scoring for α-SMA was done by Bozdogan et al. [8,25]. This study found that α-SMA immunoreactivity in the tumor was higher in infiltrative BCC, while α-SMA expression in the stroma was higher in both metatypical and infiltrative BCC. Our findings are consistent with those of earlier studies that attempted to connect SMA expression in BCC with tumor aggressiveness. The tumor cells of both aggressive and non-aggressive types of BCC showed the same level of α-SMA immunoreactivity; however, only the stroma reacts in aggressive cases. This is a useful predictor of the aggressiveness of BCC [3,6,10,26,27].

The critical role of podoplanin in tumor lymphangiogenesis is allowing lymphatic spread and metastasis of aggressive non-melanoma skin cancer [28]. Podoplanin was scored by Remmele and Stegner [29]. We demonstrated statistical significance across all BCC subtypes in the current investigation, with the adenoid subtype having the highest mean number of neoplastic cells. LVD, however, showed no such relevance. In a finding that matched earlier research, Plaza et al. discovered that 22.2% of studied BCCs expressed podoplanin, with the majority of expression in the basal layer of the tumor nest [12].

According to Tebcherani et al., only 6% of 307 examined BCCs were immunoreactive to podoplanin, with 4% of cases exhibiting a peripheral layer of tumor cell nests reactivity and 2% of cases exhibiting widespread tumor cell podoplanin expression [30]. For podoplanin immunoreactivity, LVD agreed with earlier research that revealed no significant association between podoplanin expression in BCC LVD, which might explain the very slow development and uncommon metastatic potential of BCC [28,31].

Immunoexpression of MMP-13 has been associated with malignant transformation in skin cancer, where it degrades the extracellular matrix (ECM) [32,33]. IHC scoring of MMP-13 was done in the current research [34], and we found significant MMP-13 immunoreactivity in both tumoral and stromal cells in all BCC variants. In addition, we demonstrated a greater mean in tumoral MMP-13 with metatypical BCC, whereas a higher mean in stromal MMP-13 with both infiltrative and metatypical BCC.

MMP-13 expression was also found to be not just in tumor cells but also in the cells that surround epithelial tumor cells (called stromal cells). About 65% of eyelid BCCs, as discovered by another study, had MMP-13 up-regulation in the epithelial tumoral cells located at the leading edge, which may explain the aggressive nature of this tumor [35,36].

HER2, a member of the family of epidermal growth factor receptors with tyrosine kinase activity, is expressed in various tissues where it mostly promotes carcinogenesis and excessive/uncontrolled cell proliferation. IHC scoring of HER2 was done by Florescu et al. [19,20]. Our study of HER2 in BCC found that metatypical and infiltrative BCCs had greater HER2 levels than the other subtypes.

In line with previous studies, these results showed that HER2 plays a role in how aggressive a BCC is and how it changes into different histological subtypes. In addition, several studies have claimed that HER2 is involved in the etiology of BCC [20,37].

The correlation between Immunohistochemical markers

S100 and Her2neu IHC expression proteins were significantly correlated, according to immunohistochemistry analysis, which is consistent with earlier research showing that both markers are immunoreactive in breast cancer basal cells [38,39].

Previous investigations have confirmed the link between tumoral and stromal α-SMA and HER2neu. Costa et al. found four CAF subgroups that accumulated variably in breast cancer subtypes and normal juxta-tumors [40].

Podoplanin LVD and podoplanin tumoral expression in significant difference with HER2neu agreed with previous studies showing that enhanced intratumoral reactivity of podoplanin was strongly correlated with HER2 overexpression, as measured by immunohistochemistry, and the relationship between increased intratumoral LVD and overexpression of HER2 in gastric cancer patients [41,42].

Significant relationships between tumor podoplanin and tumor MMP-13 were consistent with previous findings linking MMP-13 overexpression to advanced staging and lymph node metastases. This indicates the proteolytic activity of MMP-13 in destroying ECM and basement membrane, which promotes oral squamous cell carcinoma development and invasion [43].

Ocharoenrat et al. revealed that high levels of MMP-13 in cancer cells and stroma were significantly linked with the expression of the HER2neu protein in head and neck squamous cell carcinoma [44].

The correlation of HER2neu with itself was in line with previous research linking protein overexpression to HER2 gene amplification in breast carcinomas [45].

Limitations

This research was restricted to instances of the investigated patients, the requirement for follow-up, recurrences, degree of infiltration, and the inclusion of mixed kinds.

## Conclusions

This study showed a higher significant difference in tumoral HER2neu, stromal MMP-13, and stromal α-SMA with histological variants, notably metatypical and infiltrative BCC variants, designating these markers as proteins involved in the diagnosis of an aggressive histological pattern of BCC.

In general, the immunoreactivity for HER2neu corresponded with all other biomarkers, indicating that this marker is significant for carcinogenesis and can help predict biological aggression for effective therapy. Additionally, a significant difference was seen in the neoplastic cells for each marker S100, α-SMA, podoplanin, and MMP-13. This finding provides a prognostic clue that this neoplasm may have the capacity to differentiate in more than one way and behavior. We recommend further molecular research to evaluate the clonality of this neoplasm for studying additional biological behavior.
